# Preoperative risk factors associated with peri-operative psychiatric diagnosis in oral cancer patients

**DOI:** 10.3332/ecancer.2022.1401

**Published:** 2022-05-26

**Authors:** Arnab Mukherjee, Chitralekha Bhowmick, Shreshta Chattopadhyay, Mohamed Abdul Kathar, Moitri Bhattacharyya, Shazia Nasreen, Prateek Jain, Pattatheyil Arun, Soumitra Shankar Datta

**Affiliations:** 1 Department of Palliative Care and Psycho-Oncology, Tata Medical Centre, New Town, Rajarhat, Kolkata 700160, India; 2 Department of Head and Neck Surgery, Tata Medical Centre, New Town, Rajarhat, Kolkata 700160, India; 3 MRC Clinical Trials Unit, Institute of Clinical Trials and Methodology, University College London, London WC1V 6LJ, UK; a https://orcid.org/0000-0002-6325-7116; b https://orcid.org/0000-0002-9599-5068; c https://orcid.org/0000-0003-4363-0910; d https://orcid.org/0000-0002-8894-2670; e https://orcid.org/0000-0002-9829-8583; f https://orcid.org/0000-0003-1674-5093

**Keywords:** head and neck cancers, India, psychiatry, psycho-oncology, oral cancer

## Abstract

**Background:**

Head and neck cancers (HNCs) are one of the commonest cancers in low- and middle-income countries. There is a paucity of data on comorbid psychiatric problems associated with HNCs. The present study is aimed at reporting the pattern of psychiatric caseness in HNC patients who were referred to specialist psycho-oncology service and also investigate the predictors of psychiatric caseness in oral cancer patients.

**Methods:**

Case records of all patients with HNC referred to an integrated psycho-oncology service over 7 years (October 2011–December 2018) from a cancer hospital were analysed. All patients were assessed by a trained consultant psychiatrist and ICD-10 diagnoses were ascertained based on a clinical interview with the patients and family members. Associations of psychiatric caseness for consecutive oral cancer patients assessed by the psycho-oncology services over 2 years (January 2017–December 2018) were calculated by using univariate and multivariate statistical methods. Simple descriptive statistics of the referred patients were conducted, followed by logistic regression to find the associations of psychiatric caseness in oral cancer patients.

**Results:**

The psycho-oncology service assessed 771 HNC patients over 7 years. The commonest referrals were patients with oral cancer (75%, 558/771). For the years 2017–2018, 179 consecutive oral cancer patients were evaluated by the psycho-oncology service. Multivariate logistic regression analysis showed that being a woman (OR = 2.33; 95% CI = 1.02–5.32; *p* = 0.04); having worries about having pain in the post-operative period (OR = 2.55; 95% CI = 1.2–5.38; *p* = 0.01); worries about implications of the cancer and its treatment on the family (OR = 3.5; 95% CI = 1.19–10.57; *p* = 0.02); and longer duration of hospital stay period (OR = 1.08; 95% CI = 1.003–1.16; *p* = 0.04) were independently associated with psychiatric caseness even after controlling for confounders.

**Discussion:**

Specialist psycho-oncology services are important in the management of oral cancer patients and in addressing the mental health needs of this very vulnerable group of patients. A combination of psychoeducation, pragmatic psychological interventions and medications were used to treat these patients.

## Background

In 2020, the Global Cancer Observatory database estimated that cancers of the head and neck region constitute the second most common cancers in India [1]. It has been estimated that head and neck cancers (HNCs) constitute 25%–30% of all cancers in India [2]. In 2020 alone, there were 135,929 new patients diagnosed with oral cancer, 34,687 patients diagnosed with cancer of the larynx and 28,489 newly diagnosed patients with cancer of the hypopharynx in India [1]. Over the years, there has been an increase in awareness about cancer, along with an increase in detection rates and better outcomes of cancers, including HNCs, in different part of the world, including India [3, 4]. However, even with these advances and refinement of treatment modalities of HNCs, psychological morbidities continue to be common in these patients. Pre-existing psychiatric illness or vulnerability, the fear associated with receiving a cancer diagnosis, the need to undergo painful interventions, resultant deformities causing body image problems and financial concerns increase the risk of psychiatric disorders in HNCs [5–8].

Head and neck surgeries interfere with the motor functions, like speech, chewing and swallowing; sensory functions, like taste and smell; and social functions, like communication and expression of interest. As disfigurement due to HNC surgeries is often visible, HNCs are known to be associated with poor self-esteem, poorer self-confidence and body image difficulties when compared to less visible cancers [9]. Younger patients are more worried about disfigurement [10, 11]. Women, especially those with poor social support, have been found to be affected more often when they undergo disfiguring HNC surgeries [11]. HNC survivors with disfigurement also encounter adverse economic and social consequences and are commonly stigmatised, and research has shown that these effects persisted even after 1 year of surgery [12]. Nearly 40% of the patients reported concerns about their appearance long after the surgery [10]. Studies have shown that cosmetic rehabilitation improved patient satisfaction over time when compared with their baseline score, but this is often not a priority and referrals are infrequent to address cosmesis and appearance-related issues [13, 14]. The perceived social consequences of change in appearance is associated with higher body image concerns [15].

HNC is associated with the higher prevalence of psychological distress, even 7–11 years after treatment [16, 17]. Many of these patients did not receive psychological intervention and the focus on tumour control often takes priority in low- and middle-income countries (LMICs). HNC patients and doctors often perceive depressive and anxiety symptoms as natural responses to cancer and, as a result, patients with psychological symptoms may not report these symptoms to their oncologists, leading to both under-reporting and under-diagnosis [17]. Factors that tend to precipitate psychological distress in patients are pain and other physical symptoms, social stressors, premorbid personality factors, prognostic factors, treatment-related side effects, past history of psychiatric morbidity and treatment [18]. Studies have shown that anxiety is more common at the time of biopsy or prior to surgery [19], and people feel more depressed during the post-operative period [9]. The usual responses are worries, fear, sense of helplessness, anguish and demoralisation [6, 20]. The prevalence of depression varies across studies [19]. Studies have shown a prevalence of depression over the complete course of HNC treatment, from the time of evaluation through to recovery or palliation [21]. The severity of physical symptoms and resultant dysfunctionality, lack of emotional and social support and coping styles of individual patients were associated with increased rates of depression in HNC patients [21]. One study reported that as low as 14% of HNC patients with depression were identified and treated appropriately [18].

The aim of the present study was to report real-life data on psychiatric morbidities of HNC patients based on a 7-year long experience of providing psycho-oncology services to HNC patients in a tertiary cancer centre. The current practice in the study centre is to conduct preoperative psychiatric assessment for patients planned for HNC surgeries. The goal of this assessment is to prepare the patients for the upcoming surgery and to address their concerns so that they are less apprehensive and can cope with the treatment process better. Previous research has reported that two-thirds of the patients have reported in studies that preoperative counselling has been helpful [22, 23]. Preoperative psychiatric evaluation could also identify people at risk of post-operative psychiatric morbidities.

The scope and need of psycho-oncology services have also grown exponentially. This study examines the peri-operative psychiatric morbidities of HNC patients with a special emphasis on oral cancer from a specialist cancer centre in eastern India. Recent research on the journey of cancer patients from India has elicited complexities in the access to cancer care, emphasised the psychological journey of patients, highlighted stigma of being a cancer patient, impact of decision-making on adherence to treatment and the economic impact of cancer care alongside the modifiers to accessing cancer care [24].

## Methods

### Objectives

The first objective of the present study was to explore and describe the pattern of psychiatric morbidities in HNC patients in a specialist cancer centre. The second objective was to study the associations of psychiatric morbidities in the peri-operative period for oral cancer.

### Setting

The study was conducted in a not-for-profit cancer centre in eastern India, Tata Medical Center, Kolkata, which currently has 450 beds and state-of-the-art cancer treatment facilities. The hospital caters mainly to patients from the East Indian states of West Bengal, Jharkhand, Bihar, Assam, Sikkim, Meghalaya, Mizoram and neighbouring countries, like Bangladesh, Nepal and Bhutan. The hospital has a psycho-oncology service, established in the very first year of its inception, led by consultant psychiatrists and psychologists. The psycho-oncology department has daily out-patient services and in-patient services, both led by the two consultant psychiatrists. Most patients referred to psycho-oncology are seen on the same day of referral.

### Psycho-oncology assessment protocol for HNC patients

HNC patients were frequently referred to the department of psycho-oncology as an out-patient prior to surgery and again in the post-operative period. Patients were assessed on the same day of referral as per the existing protocol by one of the consultant psychiatrists of the department. The department of psychology, since its inception in 2011, has had a paperless documentation practice. All clinical notes were contemporaneously recorded in the electronic health records. At the end of 2016, there was a change made for the shared care protocol for patients with oral cancer as this was the commonest HNC needing psychiatric inputs. Since January 2017, all patients with oral cancers were prospectively evaluated by one of the two consultant psychiatrists during the preoperative period. Alongside a standard psychiatric assessment, the preoperative oral cancer patients were screened for the four most common psychological worries: a) worries related to the possibility of experiencing pain in the post-operative period; b) worries related to future body image problems secondary to facial disfigurement; c) worries related to direct and indirect costs of cancer care impacting finances of the family; and d) worries related to the cancer treatment disrupting family life. The psychiatric diagnosis reported in this study had taken into consideration both the preoperative psychiatric assessment and the post-operative assessment. The oral cancer patients were also referred to the psycho-oncology service in the immediate post-operative period for continuity of psychiatric care and for providing psychological support as and when appropriate. A separate contemporaneous database for oral cancer patients was started to aid in better clinical communication that was maintained by the clinical psycho-oncology fellows of the department.

### Data collection

Data for all HNC patients who had been evaluated by the department of psycho-oncology between 2011 and 2018 were examined for the purposes of this study. The records included the basic demographic details of the patient, the type and stage of cancer, medical comorbidities of the patient, type of surgery undertaken, details of the psychiatric assessment by a consultant psychiatrist, psychiatric diagnosis, if any, for the patient and the nature of interventions undertaken by the psycho-oncology service. Additionally, for the years 2017–2018, data for all oral cancer patients were maintained in the psycho-oncology departmental database. Data were entered on a predetermined proforma. In view of the retrospective nature of the study, waiver of consent was obtained from the institutional ethics committee as per institutional policy (IEC Protocol Waiver No – EC/WV/TMC/012/19).

### Data analysis

The data were analysed using Statistical Package for the Social Sciences 25. Univariate analysis included chi-squared test for categorical variables and *t*-test for continuous variables. Multivariate logistic regression analysis was carried out for patients with oral cancer, using the dichotomous variable of psychiatric caseness as the intendant variable and other variables as predictor variables that were significant in univariate analysis.

## Results

Over the period of the study, the Department of Psycho-oncology had evaluated 771 HNC patients (Table 1). Majority of them were men (72%, 555/771) and had been diagnosed to have oral cancers (75%, 558/771) that included cancer of the tongue, buccal mucosa, alveolus, lip, retromolar trigone, floor of mouth and hard palate. Of these patients, primary closure was carried out for 26.6% (205/771) and reconstruction for 59.1% (456/771). 14.3% of the patients seen by psycho-oncology and HNC service were inoperable as they presented with late stages. Tracheostomy was performed in 44.1% of the patients (240/771) and it was not needed in 55.9% of the patients (431/771). Psychiatric diagnoses were based on the assessments made: immediate preoperative psychiatric assessment and immediate post-operative psychiatric assessment. All patients were assessed by one of the two consultant psychiatrists with special expertise in psycho-oncology. Over the years, around one-third of the patients assessed by the psycho-oncology service were found to have no psychiatric diagnosis.

Since 2017–2018, all patients with oral cancer were assessed preoperatively by one of the consultant psychiatrists who led the psycho- oncology service. The commonest cancers within the oral cavity (Table 2) included tongue cancer (41.3%, 74/179) and cancer of the buccal mucosa (31.8%, 57/179). More than half (53%, 95/179) of the patients with oral cancer had qualified for a psychiatric diagnosis. The commonest psychiatric comorbidities (Table 3) for peri-operative oral cancer patients were anxiety disorders (26.3%, 47/179), adjustment disorders (12.8%, 23/179) and depression (6.1%, 11/179). Univariate analysis showed that the following factors were predictive of peri-operative psychiatric diagnosis: being a woman, having worries about experiencing pain in the post-operative period, having worries regarding body image in the post-operative period and worries about the negative impact of the treatment on the family (Table 4). Several other factors, such as distance travelled from home to the hospital, age of the patient, occupation of the patient, stage of the disease and presence of tracheostomy tube, were not associated with increased psychopathology in HNC patients. Multivariate logistic regression analysis (Table 5) showed that being a woman (OR = 2.33; 95% CI = 1.02–5.32; *p* = 0.04); having worries about experiencing pain in the post-operative period (OR = 2.55; 95% CI = 1.2–5.38; *p* = 0.01); worries about implications of the cancer and its treatment on the family (OR = 3.5; 95% CI = 1.19–10.57; *p* = 0.02); and longer duration of hospital stay (OR = 1.08; 95% CI = 1.003–1.16; *p* = 0.04) were independently associated with psychiatric caseness even after controlling for confounders. Surprisingly, worries about potential body image and deformity was not associated with psychiatric caseness.

## Discussion

Surgeries for oral cancer are often associated with significant disfigurement, physical and emotional pain and resultant emotional distress. The present study on psychiatric morbidity in patients with oral cancer showed that being a woman, having preoperative worries about pain, having preoperative worries about impact of the cancer treatment on the family and longer duration of hospital stay were independently associated with psychiatric diagnosis in the peri-operative period. The strengths of the present study include a relatively large sample size, evaluation being conducted by a trained consultant psychiatrist with special interest in psycho-oncology and the study being set in the LMIC that contributes the greatest number of HNC patients globally. Having a dedicated psycho-oncology service working closely with HNC services and having a standardised preoperative psychiatric evaluation protocol for HNC patients made the assessments uniform. The weaknesses of the study were a) the cross-sectional nature of the study and b) being conducted in a single centre.

The present study on oral cancer patients showed that being a woman was associated with psychiatric morbidity. This follows a similar trend around the world [25, 26] and is in line with the findings of increased mood disorders in women around the world [27]. There may be additional social factors that make women with cancer more vulnerable in LMICs based on the existing gender gap [28] and inequality in access to care [29, 30]. Many patients expressed significant worries about the implications of their illness and treatment on their family. Our study showed that preoperative worrying of patients about their family was associated with a psychiatric diagnosis. They were often anxious that their illness and treatment would impact the job of their caregivers, the education of their children and the overall financial security of the family. Previous research from the same centre showed that patients had worries related to the impact of the cancer on them and their families due to the requirement of family members staying back from work due to the diagnosis of cancer; occasional inability of family members to continue with their education; and being stigmatised due to their diagnosis in their day-to-day lives [24]. Most of the cancer care in India is funded by the patient and their families and researchers have highlighted the lack of infrastructure, paucity of trained resources and escalated costs of cancer care in India [31].

Body image is a subjective perception about one’s own appearance, and is often modified by the reactions elicited from others [32]. People with oral cancers often experience body image disturbance secondary to disfigurement related to surgery. Earlier research on preoperative evaluations showed more than three-quarter of the people with oral cancers have concerns about their body image [33]. Body image disturbances are known to lead to self-stigma, social isolation, difficulty in reintegrating into previous job, intimacy with partner and psychological morbidities [12]. However, a surprising finding in our study was that perceived worries about potential body image problems were not associated with more psychiatric problems. This could be due to the fact that we studied the patients in the immediate pre- and post-operative periods, and the problems of body image may evolve over time and require long-term follow-up. Pain and physical symptoms remain important predictors for depression [34] and are associated with psychological morbidities in oral cancer patients. Earlier research has shown that presurgical anxiety about pain remains an important predictor for post-surgical intensity of pain and psychological morbidity [34–36]. Experiencing physical pain even after completion of treatment was found to be associated with depressive and anxiety symptoms in previous studies [37].

Our study found longer hospital stay was associated with increased psychiatric problems as reported by studies from other parts of the world [38]. Prolonged admission in the post-operative phase is almost always due to multiple medical morbidities and more intense monitoring of medical parameters. Earlier work on patients with oral cancer from the UK had shown that patients were psychologically relieved when the monitoring was stepped down from hourly to 4 hours [39]. The association of psychiatric diagnosis with longer duration of admission is thus not surprising.

The role of psycho-oncology services is extremely valuable in HNCs. Preoperative evaluation of anxiety and common worries, preparedness for treatment, social stressors, personality factors, coping resources and pre-existing psychiatric morbidities can help the treating team to assess the presence of or anticipate risk of psychological morbidity in a patient. It can provide valuable clinical information about the patient’s coping ability. Unaddressed psychiatric morbidities may interfere with the ability of the patient to engage with oncology services. This study reports the findings of the preoperative psychiatric evaluation for people with oral cancers. The common interventions undertaken by the psycho-oncology service included a mental health assessment of the patient in the context of the medical condition, planning appropriate psychiatric care and coordinating with the surgical service and the intensive care unit. The profile of a typical HNC patient who qualifies for various psychiatric diagnoses, reasons for referrals, goals of mental health care in the peri-operative period and interventions offered are summarised for the reader in Table 6. The value of timely interventions cannot be over emphasised.

## Conclusion

HNCs are one of the commonest cancers in LMICs and require multidisciplinary care. Psychiatric comorbidities are common and are best managed when referred to a specialist psycho-oncology service early on. The various mental health conditions may require specialist mental health inputs and are best managed jointly with HNC surgical oncologists. There is very little systematic data on this group from LMICs and further research is required in the future.

## Conflicts of interest

The authors declare that they do not have any conflicts of interest.

## Funding

The work was not supported by any external funding.

## Figures and Tables

**Table 1.** Characteristics of HNC patients seen by psycho-oncology services from 2011 to 2018.

Variables	Mean + SD *n* = 771	Range
**Age**	54.9 + 13.2	13–94
	**Frequency**	**Percentage**
**Gender** Male Female	555 216	72.0 28.0
**Surgical intervention** Primary closure only was needed Reconstruction undertaken Inoperable (surgery not done)	205 456 110	26.6 59.1 14.3
**Tracheostomy** Tracheostomy done Tracheostomy not needed	340 431	44.1 55.9
**Site** Tongue Buccal mucosa Alveolus Pharynx Larynx Thyroid Hard palate Nasal cavity/paranasal sinuses Lip Floor of mouth Salivary gland Retromolar trigone Others	220 179 106 66 57 44 16 16 14 13 13 10 17	28.5 23.2 13.7 8.6 7.4 5.7 2.1 2.1 1.8 1.7 1.7 1.7 2.04
**Psychiatric diagnosis** No psychiatric morbidity Adjustment disorder Depression Delirium Anxiety Substance misuse Organic mood/psychotic disorders Schizophrenia Bipolar affective disorder Other psychiatric disorders Other psychotic disorder	234 124 115 92 92 60 16 19 11 9 2	30.3 16.1 14.9 11.9 11.9 7.8 2.1 2.5 1.4 1 0.3

SD: Standard deviation

**Table 2.** Characteristics of patients with oral cancer evaluated prospectively by psycho-oncology services in 2017–2018 .

Variables	Mean (*n* = 179)	Standard deviation	Median	IQR
**Age (years)**	54.4	12.4	54	45–64
	**Frequency (*n* = 179)**		**Percentage**	
**Gender** Male Female	134 45		74.9 25.0	
**Marital status** Married Single Widowed/widower	165 10 4		92.2 5.6 2.2	
**Occupation** Service Own business Homemaker Professional Retired Unskilled labourer Unemployed Not known	52 38 36 12 7 7 2 25		29.1 21.2 20.1 6.7 3.9 3.9 1.1 14	
**Stage of illness** Early (Stage 1 and 2) Advanced (Stage 3 and 4)	60 119		33.5 66.5	
**Site** Tongue Buccal mucosa Alveolus Hard palate Retromolar trigone Lip Floor of mouth	74 57 35 7 3 2 1		41.3 31.8 19.6 3.9 1.7 1.1 0.6	

IQR: Interquartile range

**Table 3.** Peri-operative psychiatric diagnosis of patients with oral cancer.

Psychiatric diagnosis (*n* = 179)	Frequency	Percentage
No psychiatric morbidity	84	46.1
Anxiety	47	26.3
Adjustment disorder	23	12.8
Depression	11	6.1
Substance misuse	10	5.6
Schizophrenia	3	1.7
Dementia	1	0.6

**Table 4.** Univariate analysis: associations with psychiatric diagnosis as diagnosed by ICD-10 in peri-operative HNC patients.

Variables	Patients with psychiatric diagnosis (*n* = 95)	Patients with no psychiatric diagnosis (*n* = 84)	Mann–Whitney *U*	*p*
Age (median, IQR, range in years)	54, IQR 45–61, range 25–80 years	55, IQR 45–65, range 31–82 years	3.8	0.61
Duration of hospital stay (median, IQR, range in days)	9, IQR 6–12, range 2–36 days	8, IQR 5.25–10, range 1–21 days	3,358	0.07
Time since cancer diagnosis (median, IQR, range in months)	9, IQR 2–21, range 1–1,480 months	10.5, IQR 4–21.75, range 0–231 months	3,671	0.36
			***χ* ^2^**	***p* value**
**Gender** Male Female	63 32	71 13	7.854	0.005
**Occupation** Service/employed Homemaker Own business Professional Unskilled Retired Unemployed Not known	30 27 17 5 3 2 1 10	22 9 21 7 4 5 1 15	12.78	0.07
**Stage of illness ** Early Advanced	33 62	27 57	0.135	0.714
**Tracheostomy** Tracheostomy done Tracheostomy not needed	67 28	58 26	0.046	0.83
**Worries related to pain ** Yes No **Worries related to body image** Yes No **Worries related to implication of illness and treatment on family** Yes No **Worries related to financial constraints** Yes No	41 54 35 60 35 60 33 62	1 67 19 65 16 68 19 65	10.692 4.281 6.929 3.176	0.001 0.039 0.008 0.075

**Table 5.** Multivariate analysis: associations of psychiatric caseness in oral cancer patients in the peri-operative period.

Variable	*B*	Sig	OR (95% CI)
Gender	0.85	**0.04**	2.33 (1.02–5.32)
Worries related to pain	0.935	**0.01**	2.55 (1.2–5.38)
Worries related to future body image problems	0.55	0.9	1.06 (0.44–2.55)
Worries related to future implication of illness and treatment on family	1.26	**0.02**	3.5 (1.19–10.57)
Worries related to future financial constraints	−0.46	0.39	0.631 (0.22–1.81)
Duration of hospital stay	0.76	**0.04**	1.08 (1.003–1.16)

*p* < 0.05

**Table 6.** The prototypes of patients with HNCs jointly managed by psycho-oncology services.

Typical demographic profile	Psychiatric diagnosis	Usual reasons for referrals and goals of treatment	Common interventions offered by psycho-oncology
35–45-year-old male with no past psychiatric history presenting with anxiety	Adjustment disorder	Reason: Anxiety and difficulties in coping with physical symptoms Goal: Promote adaptation	Psychological intervention to reduce distress and improve coping When required short-term low dose antidepressants (escitalopram, mirtazapine) along with occasional clonazepam/zolpidem was the preferred pharmacological intervention
30–60-year-old male/female	Anxiety disorder	Reason: Anxiety symptoms Goal: Reduce anxiety and improve coping	Pharmacological interventions: SSRIs (Selective Serotonergic Re-uptake inhibitors) e.g. escitalopram, sertraline. Psychological interventions for anxiety disorder and relaxation training.
30–45-year-old man with comorbid alcohol dependence presenting with restlessness	Alcohol dependence	Reason: Evaluate and manage restlessness and complicated withdrawal Goal: Prevent or manage potential complicated alcohol withdrawal	Detoxification included physical examination, regular mental state examination and monitoring of active withdrawal symptoms. The patients were started on tapering doses of benzodiazepines alongside supplementation with thiamine to prevent Korsakoff’s syndrome. Psychological interventions: Deaddiction treatment after immediate post-operative period was aimed at preventing relapse of substance misuse.
45–55-year-old man/woman with clinical depression	Depression	Reason: Patient appearing depressed and low in mood Goal: Assess low mood, conduct risk assessment for completed suicide	Pharmacological, psychological or combined treatment for depression Suicide risk assessment and management
30–55-year-old man/woman with previous diagnosis of severe enduring mental illness	Schizophrenia Bipolar disorder	Reason: Assess pre-existing serious psychiatric illnesses. Goal: Manage pre-existing serious psychiatric illnesses and optimise medications in the peri-operative period.	Optimising the pharmacological treatment of schizophrenia, delusional disorder and severe mood disorders. Psychological management of mental health condition done.
Over 60-year-old man/woman with forgetfulness/early dementia	Mild cognitive impairment Dementia	Reason: Assess cognitive dysfunction and manage appropriately. Goal: Devise a care plan that addresses the needs of the patient and helps him/her engage with oncology services.	Conducting assessment for potentially reversible causes of dementia and quantifying the degree of cognitive impairment. Coordinating the care with nurses and medical consultants to provide care to the elderly person with cognitive problems.
